# Closed-Loop Nuclear Magnetic Resonance Gyroscope Based on Rb-Xe

**DOI:** 10.1038/s41598-020-59088-y

**Published:** 2020-02-10

**Authors:** Ke Zhang, Nan Zhao, Yan-Hua Wang

**Affiliations:** 1 Beijing Computational Science Research Center, Beijing, 100193 China; 2 Shan Xi University, Tai Yuan, 030006 China; 3 Graduate School of China Academy of Engineering Physics, Beijing, 100193 China

**Keywords:** Applied physics, Optical physics

## Abstract

Nuclear magnetic resonance gyroscopes have the potential to outperform other kinds of gyroscopes with the merits of high precision, small volume, low consumption. Here we present a closed-loop NMRG system based on the spin-exchange optical pumping of Rb-Xe. We have established a theoretical model for the closed-loop NMRG system and obtained the transfer function. The step response, frequency response of the closed-loop NMRG system are calculated through the transfer function. We also have studied the influence of closed-loop parameters for the performance of NMRG experimentally, involving step response, frequency response, and sensitivity. The experimental results are in good agreement with the theoretical data. Our work is promising in improving the performance of NMRG in the future.

## Introduction

Gyroscopes measure the angle or angle rate of the object with respect to the inertial space^1,2^, which has key applications in aerospace, aviation, and navigation fields. With the rapid development of atomic manipulation technology, nuclear magnetic resonance gyroscopes (NMRGs) have been developed over the past decades. The NMRGs have the potential advantages of high precision, compact size, acceleration insensitivity, and low power consumption^3–5^, which have a significant influence in future inertial field^6,7^. The NMRGs accomplish rotation measurement through measuring the shift of precession frequency of noble gas nuclei^5,8^ in an applied magnetic field. Large scale research of NMRGs have been done in 1960s and 70s^9–12^. As of the early 1980s, Singer-Kearfott company realized the NMRG based on ^199^Hg, ^201^Hg, the angle random walk (AWK) reached 0.05°/$\sqrt{{\rm{h}}}$, and the bias stability is 0.02°/h^9,10^. Besides, the spin-exchange optical pumping (SEOP) technique of alkali metal atoms and noble gas nuclei is used in NMRG^13^. The NMRGs based on this approach achieved a bias instability near 0.01°/h and an ARW of 0.002°/$\sqrt{{\rm{h}}}$^14^. Recently, a new type of NMRG based on K-^3^He is proposed without spin-exchange relaxation, achieved an ARW of 0.002°/$\sqrt{{\rm{h}}}$ and a bias instability of 0.04°/h^8^. Other researches for NMRG are found in refs. ^4,15,16^.

Although large scale of researches have been done for NMRG. However, the bias stability of NMRG is still lower than other kinds of gyroscopes, such as atomic interference gyroscope, fiber optic gyroscope. There are many factors to influence the bias stability of NMRG. Working with closed-loop state is an effective method experimentally. When the closed-loop control parameters are properly chosen, the noise of the system will be suppressed largely, especially for the low frequency noise. Then the precision of the NMRG will be improved. Besides, closed-loop system will have higher measurement range than open-loop system. Thus, the research of closed-loop NMRG and the selection of closed-loop control parameters will be very important for improving the performance of NMRG.

In this paper, we have given a systematic research of closed-loop NMRG and a theoretical model is established. The closed-loop NMRG system involves open-loop NMRG, filter, and phase-locked loop (PLL). We describe the dynamics of nuclear spins near the resonant point through Bloch equation, then obtain the transfer function of open-loop NMRG. Based on the automatic control theory, the transfer function of closed-loop transfer function is also obtained. Then we calculate the step response, frequency response of the closed-loop NMRG based on the closed-loop transfer function theoretically. Due to the controlled parameters of PLL is very significant for the closed-loop system, which will determine the sensitivity and bandwidth of closed-loop NMRG. Thus, we also study the step response, frequency response, noise spectrum of inertial signal for closed-loop NMRG system with different controlled parameters of PLL experimentally, namely the parameters P and I. The experimental results are in good agreement with the theoretical data. We conclude that the bandwidth of closed-loop NMRG will increase with the value of P, and the noise will also be increased (i.e. the sensitivity of NMRG will be lower). The sensitivity and bandwidth of closed-loop NMRG cannot be improved simultaneously. For a fixed value of P, I has an optimal value which is the critical value of preventing the system from oscillating.

## Experimental Setup and Theoretical Description

**Figure 1:**
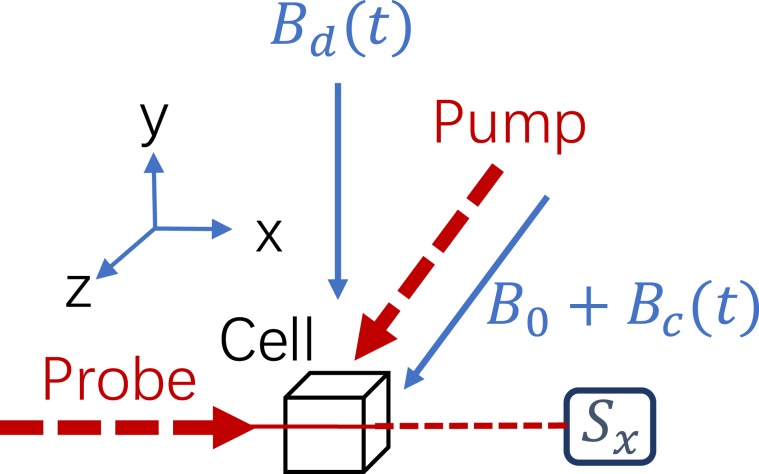
The schematic of basic principle of nuclear magnetic resonance gyro. The pump beam polarize the Rb electronic spins along *z* direction. Then the Xe nuclei are polarized by the electronic spins through spin-exchange collisions. A radio frequency magnetic field (RF field) ${B}_{d}(t)$ along *y* direction is utilized to drive the nuclear spins deviate from *z* axis and precess about *B*_0_ with frequency *γB*_0_. The precessing nuclear spins will generate transverse oscillating magnetic fields. The electronic spins along *z* direction are influenced by the nuclear magnetic fields, then will have projection *S*_*x*_ along the probe direction *x*. We detect the electronic spins with probe beam, then deducing the nuclear precession frequency. When the gyroscope rotate about *z* axis with angular velocity ${\omega }_{r}$, the precession frequency of nuclear spins will be $\gamma {B}_{0}+{\omega }_{r}$. Thus, the shift of nuclear precession frequency is corresponding to angular velocity ${\omega }_{r}$.

A schematic of basic principle of NMRG is shown in Fig. 1. Rubidium, isotopically enriched Xe, along with N_2_ gas, are contained in a cubic glass cell with the side of 1.5 cm. N_2_ is used to eliminate radiation trapping and prevent wall collisions. A boronnitride oven is used to heat the vapor cell to 110 °C. The alkali metal electronic spins are optically pumped with circularly polarized pump laser propagating parallel to the bias magnetic field *B*_0_ and modulation magnetic field ${B}_{c}\,\cos \,{\omega }_{c}t$, which is along the sensitive rotation axis *z* for NMRG. The power of pump laser is 165 mW. We set the bias magnetic field to 19.6 *μ*T, and *B*_*c*_ = 50 *μ*T. The modulation frequency ${\omega }_{c}=93\,{\rm{kHz}}$, resonant with ^85^Rb Larmor precession frequency. Polarized alkali metal electronic spins transfer angular momentum to the noble gas nuclei via spin-exchange collisions^17,18^. After the Xe nuclei undergo tens of seconds of spin-exchange collisions with polarized Rb, Xe nuclei will reach a steady-state polarization. Then the polarized Xe nuclei are driven by a radio frequency magnetic field ${B}_{d}(t)$ (RF field) which oscillates at a frequency near the Larmor precession frequency of Xe nuclei. The RF field will tip the Xe nuclear spins deviate from the initial direction *z*, and then precess about the bias field *B*_0_. The precessing nuclear spins will produce transverse (*x* and *y*) oscillating magnetic fields, sensed by alkali metal electronic spins. When transverse magnetic fields (*B*_*x*_, *B*_*y*_) are nonzero, the electronic spins along *z* direction will have projection *S*_*x*_ along *x* axis, and will be detected by a linearly polarized light propagating along *x* direction with the power of 4 mW. Thus we can obtain the nuclear magnetic fields from the photo detector (PD) signal, then deduce the nuclear precession frequency.

**Figure 2:**
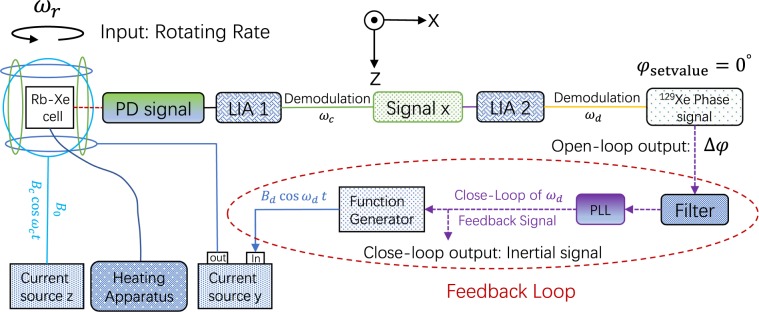
The schematic of closed-loop NMRG apparatus. The PD signal is demodulated with ${\omega }_{c}$ and specific phase, obtaining the signal which is sensitive to *B*_*x*_, used to measure the nuclear magnetic field. Demodulate the measured nuclear magnetic field signal with ${\omega }_{d}$, the amplitude and phase of nuclear oscillating magnetic field are obtained. When the frequency ${\omega }_{d}$ of RF field is resonant with the observed Xe Larmor precession frequency, the phase of measured nuclear magnetic field is zero, which is also the working point of NMRG. When the apparatus rotate by *z* axis with angular velocity ${\omega }_{r}$, the observed nuclear precessing frequency will shift ${\omega }_{r}$, lead to the phase error signal Δ$\phi$ is nonzero. The phase error signal behave as the input of phase-locked loop (PLL), then the output of PLL is fed back to calibrate the frequency ${\omega }_{d}$ of radio frequency field resonant with observed nuclear precessing frequency $\gamma {B}_{0}+{\omega }_{r}$, i.e. ${\omega }_{d}=\gamma {B}_{0}+{\omega }_{r}$. Then the variation of frequency ${\omega }_{d}$ will represent the rotation rate ${\omega }_{r}$ of apparatus about the bias magnetic field.

Figure 2 shows the schematic of closed-loop NMRG apparatus, which include the open-loop NMRG system and feedback part. The PD signal is the electronic spin signal *S*_*x*_, which is sensitive to nuclear transverse magnetic fields *B*_*x*_, *B*_*y*_. After demodulating the PD signal with ${\omega }_{c}$ and different phases, we obtained two signals, which is sensitive to *B*_*x*_ and *B*_*y*_ separately. The signal sensitive to *B*_*x*_ (signal *x* in Fig. 2) is utilized to measure the nuclear oscillating magnetic field. If the signal *x* is demodulated with ${\omega }_{d}$, one can obtain the amplitude and phase $\phi$ of nuclear magnetic field signal. When ${\omega }_{d}$ is resonant with the observed precession frequency of Xe nuclei, the amplitude of nuclear magnetic field reach to maximum value and the phase $\phi$ is zero, which is also the working point of NMRG. For the open-loop NMRG system, the frequency of RF field satisfy the condition of ${\omega }_{d}={\gamma }_{{\rm{Xe}}}{B}_{0}$. Thus, ${\omega }_{d}$ is resonant with the observed precession frequency ${\gamma }_{{\rm{Xe}}}{B}_{0}$ of Xe nuclei when ${\omega }_{r}=0$, phase $\phi$ is zero. When the apparatus rotates about *z* axis with angular velocity ${\omega }_{r}\ne 0$, the observed precession frequency of Xe nuclei will shift ${\omega }_{r}$, i.e. $\gamma {B}_{0}+{\omega }_{r}$. Then the NMRG system will be nonresonant, the frequency of RF field ${\omega }_{d}$ is not equal to the observed precession frequency of nuclear spins, the phase $\phi$ will be nonzero. Thus, the angular velocity ${\omega }_{r}$ can be deduced through the phase variation Δ$\phi$ for the open-loop NMRG system. As is shown in Fig. 2, when the apparatus rotate by the $z$ axis with angular rate ${\omega }_{r}$, there will induce the phase error signal Δ$\varphi$. The closed-loop feedback part will ensure that the system always stay in resonant state, which include filter, phase-locked loop (PLL). The filter constant and order of the filter is setted to $\tau \,=\,\mathrm{5\ }{\rm{ms}}$ and $k\,=\,8$ respectively. When the rotating rate ${\omega }_{r}\ne 0$, Δ$\phi$ will be nonzero. Then Δ$\phi$ pass through the filter, behaving as the input signal of PLL. The output signal of PLL is fed back to calibrate the frequency ${\omega }_{d}$ of radio frequency (RF) magnetic field, which enable that the frequency ${\omega }_{d}$ is resonant with observed nuclear precession frequency $\gamma {B}_{0}+{\omega }_{r}$, namely the phase error signal is zero. Thus the input of rotating rate ${\omega }_{r}$ will be compensated to the frequency ${\omega }_{d}$ of RF magnetic field through the closed loop feedback part. As long as the bias magnetic field ${B}_{0}$ is steady, the shift of the frequency ${\omega }_{d}$ of RF magnetic field in closed-loop system will be precisely equal to the inertial signal, also the rotating rate ${\omega }_{r}$ of carrier.

In the following, we consider the dynamics of ${\rm{Xe}}$ nuclei near the working point of NMRG. Ignoring the magnetic field generated by electronic spins, the spin dynamics of ${\rm{Xe}}$ nuclei can be well modeled by Bloch equation. As is shown in Fig. 1, a bias magnetic field ${B}_{0}$ is applied along $z$ axis and a radio frequency magnetic field ${B}_{d}sin({\omega }_{d}t-\phi )$ is applied along $y$ direction, whose amplitude and frequency are ${B}_{d}$ and ${\omega }_{d}$. The phase difference between the detected signal and RF magnetic field is denoted by $\varphi$. Then the Bloch equation for describing the precession of Xe nuclei is^19^

1 $$\frac{d{{\bf{M}}}^{{\bf{n}}}}{dt}=-\,{\boldsymbol{\Omega }}\times {{\bf{M}}}^{{\bf{n}}}-\Gamma \cdot {{\bf{M}}}^{{\bf{n}}}+{{\bf{R}}}_{se},$$

where **M**^**n**^ represents the nuclear polarization vector, and **Ω** is the whole angular velocity of Xe nuclei, namely the observed precession frequency. The spin exchange pumping rate is **R**_*se*_. Since the Xe nuclei are polarized by Rb electronic spins along *z* axis via spin-exchange optical pumping process, thus **R**_*se*_ is also along *z* axis. The relaxation process is depicted by ${\Gamma }_{2}(\hat{x}\hat{x}+\hat{y}\hat{y})+{\Gamma }_{1}\hat{z}\hat{z}$. ${\Gamma }_{1}$ and ${\Gamma }_{2}$ represent longitudinal and transverse relaxation respectively.

Based on the steady state solution of ref. ^19^ for Eq. 1, we obtain

2 $${T}_{2}\frac{d\phi }{dt}+\phi ={T}_{2}\Delta .$$

where $\Delta ={\Omega }_{z}-{\omega }_{T}$, and ${\omega }_{T}$ is the nuclear Larmor precession frequency. Here ${T}_{2}=1$/${\Gamma }_{2}$ represents the transverse relaxation time of nuclear spin. When the apparatus rotate about *z* axis with angular velocity ${\omega }_{r}$, the whole angular velocity Ω_*z*_ along *z* direction will be ${\omega }_{T}+{\omega }_{r}$, i.e. $\Delta ={\omega }_{r}$. Thus Ω is also the input angular velocity ${\omega }_{r}$ of NMRG. According to the automatic control theory, the NMRG system belong to the first order system.

We solve the Eq. 2 with Laplace transform, then

3a $$\phi (s)={\int }_{0}^{\infty }\,\phi (t){e}^{-s(t)}\,{\rm{d}}t,$$

3b $$\Delta (s)={\int }_{0}^{\infty }\,\Delta (t){e}^{-s(t)}\,{\rm{d}}t,$$

The Laplace transform of Eq. (2) will be

4 $$s\varphi (s)-\phi (0)=-\,\frac{1}{{T}_{2}}\phi (s)+\Delta (s),$$

Since that the working point of NMRG system is $\phi =0^\circ$. Thus, the initial value of phase $\phi$ is zero, i.e. $\phi (0)=0^\circ$. With the initial condition, we can obtain

5 $$\phi (s)=\frac{{T}_{2}}{1+{T}_{2}s}\Delta (s)=G(s)\Delta (s),$$

where $G(s)=\frac{{T}_{2}}{1+{T}_{2}s}$ is the transfer function of open-loop NMRG system. From Eq. 5, we know that Δ(*s*) (i.e. ${\omega }_{r}$) is the input of open-loop NMRG system, and $\phi (s)$ (i.e. Error signal Δ$\phi$ in Fig. 2) is the output response signal of open-loop NMRG system.

For the whole closed-loop NMRG system in Fig. 2, the system include not only the open-loop NMRG part but also the feedback loop part. The transfer function of filter is

6 $$F(s)=\frac{1}{{(1+\tau s)}^{k}},$$

where $\tau$, *k* are the filter constant and filter order respectively. The transfer function of PLL controller is

7 $$C(s)={k}_{p}+{k}_{i}/s+{k}_{d}\ast s,$$

where *k*_*p*_, *k*_*i*_, *k*_*d*_ are the P (Proportion), I (Integral), D (Differential) parameters for PLL controller. Based on the automatic control theory, the transfer function of closed-loop NMRG system shown in Fig. 2 is

8 $$H(s)=\frac{G(s)F(s)C(s)}{1+G(s)F(s)C(s)}.$$

In the theoretical calculation process, we utilize the MATLAB code’feedback’ and *G*(*s*), *F*(*s*), *C*(*s*) to calculate the transfer function *H*(*s*). The theoretical curves of step response and frequency response are obtained through the transfer function *H*(*s*) with MATLAB code ‘step’ and ‘bode’ respectively.

## Results

For a controlled system, we usually test the performance based on the step response, frequency response, power spectrum density (PSD) of measurement signal. The step response describes how the system response to a direct current (DC) signal input, while the frequency response describes how the system response to an alternating current signal input, which determine the bandwidth of controlled system. The PSD describes the noise spectrum of measurement signal, which determine the sensitivity of controlled system. Here we observe the step response, frequency response, and noise spectrum of inertial signal with different closed-loop parameters (P, I, D) for closed-loop NMRG system. Since that the closed-loop controlled parameter D is applied for the system which sense the variation of environment slowly, for example, the temperature control system. Thus, for the closed-loop NMRG system, the function of D is not apparent, thus the value of D is setted to zero in our experiment.

In the following, we set the parameters based on experiment for theoretical calculation. The transverse relaxation time of nuclear spins is ${T}_{2}=11.1\,{\rm{s}}$. The filter constant and filter order is $\tau =5\,{\rm{ms}}$, $k=8$ respectively. Based on Eqs. 5, 6 and 7, we obtain the transfer function *G*(*s*), *F*(*s*), *C*(*s*). Based on Eq. 8 and MATLAB code‘feedback’, we obtain the closed-loop transfer function *H*(*s*). Then we use MATLAB code ‘step’, ‘bode’ and transfer function *H*(*s*) to calculate the theoretical curves of step response and frequency response respectively.

**Figure 3:**
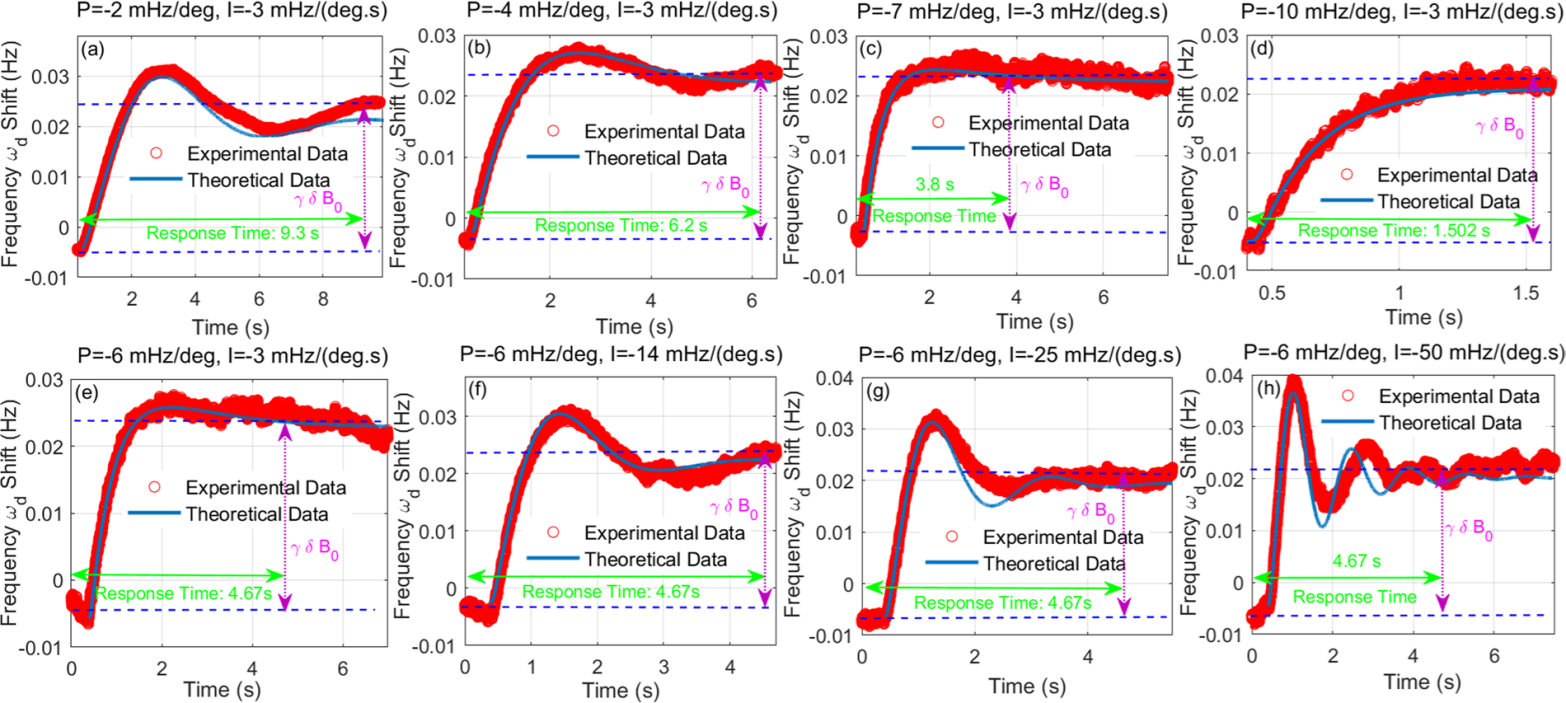
The step response signal of closed-loop NMRG system with the bias magnetic field *B*_0_ increased by 3 nT suddenly, for various values of P, I. For (**a**–**d**), the value of I is fixed on −3 mHz/(deg.s). For (**e**–**h**), the value of P is fixed on −6 mHz/deg. P, I are the control parameters of PLL controller. The variation $\delta {B}_{0}=3\,{\rm{nT}}$ of bias magnetic field is utilized to simulate the input of rotating rate ${\gamma }_{{\rm{Xe}}}\delta {B}_{0}$ for closed-loop NMRG. The blue solid curve represents the theoretical data calculated through the transfer function $H(s)$ for closed-loop NMRG system. The red circles are the observed experimental signals. The blue dashed lines represent the initial and final state of inertial signal, then the difference is the rotating rate ${\gamma }_{{\rm{Xe}}}\delta {B}_{0}$. The corresponding time is the response time of closed-loop system for the input signal ${\gamma }_{{\rm{Xe}}}\delta {B}_{0}$.

Figure 3(a–d) show the step response signal of closed-loop NMRG system for different P parameters of PLL, with the value of parameter I fixed on −3 mHz/(deg.s). The value of parameter P is −2 mHz/deg, −4 mHz/deg, −7 mHz/deg for Fig. 3(a–d) respectively. The data of longitudinal axis is the shift of frequency ${\omega }_{d}$, also the inertial signal. We utilize the variation *δB*_0_ of bias magnetic field to simulate the rotate rate *γδB*_0_ of the carrier. The observed experimental signals in Fig. 3 are obtained when the bias magnetic field have a 3 nT variation suddenly for closed-loop NMRG system, which are the red circles in Fig. 3. The blue solid line is the theoretical data calculated through the transfer function $H(s)$. The lower dashed blue line represents the initial state of inertial signal, i.e. the working point of closed-loop NMRG system. The upper blue dashed line represents the final state of inertial signal after the input of rotating rate *γδB*_0_. Thus, the difference between these two dashed blue lines correspond to *γδB*_0_. The response time is defined by the time that system vary from initial state to final state, which have been illustrated in Fig. 3 with green lines. It is easily found that the time that system response to the rotating rate *γδB*_0_ decreases with the value of P increases, from around 9.3 s to 1.5 s when P increases from −2 mHz/deg to −7 mHz/deg. It can be qualitatively understood that the system response to the input signal much faster with larger value of P, namely that the bandwidth of system is higher. Nevertheless, it also can be seen that the noise of the inertial signal become higher with larger value of P, which will influence the final sensitivity of NMRG system.

Figure 3(e–h) show the step response signal of closed-loop NMRG system for different values of I, with the value of parameter P fixed on −6 mHz/deg. The value of I is −3 mHz/(deg.s), −14 $\text{mHz}/(\deg \text{.s})$, −25 mHz/(deg.s), −50 mHz/(deg.s) for Fig. 3(e–h) respectively. We also obtain the experimental signal through increasing the bias magnetic field by 3 nT. With the value of I increases, the response time of system is hardly variational, about 4.67 s. Besides, the system will oscillate before it stay in next resonant state, the oscillation frequency increases with the value of I increases. The noise of inertial signal is also hardly variational, which also means that the sensitivity of closed-loop NMRG will be unchanged.

**Figure 4:**
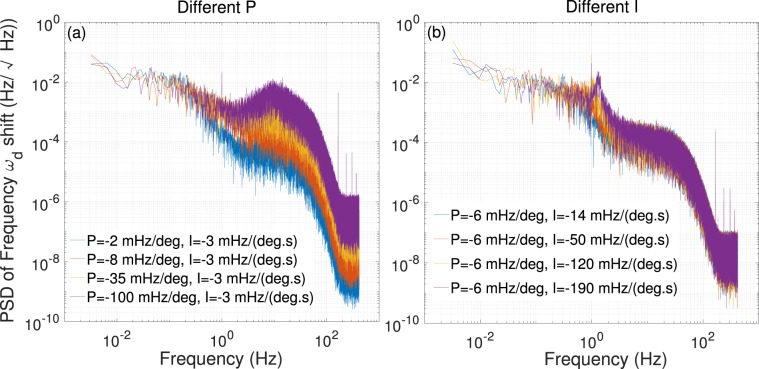
The power spectrum density (PSD) of frequency ${\omega }_{d}$ shift for the closed-loop NMRG system. (**a**) The measured PSD for different values of P, the value of I is fixed on −3 mHz/(deg.s). (**b**) The measured PSD for different values of I, the value of P is fixed on −6 mHz/deg.

Figure 4 shows the noise spectrum of inertial signal for different values of P and I parameters. The observed noise spectrum for different values of P is shown in Fig. 4(a), the value of I is fixed on −3 mHz/(deg.s). It can be seen that the low frequency noise is not variational when the value of P increases. When the noise frequency is higher than 0.1 Hz, the noise level increases with the value of P increases, which is also in agreement with the quantative conclusion obtained in Fig. 3(a–d). It means that the sensitivity of closed-loop NMRG will be lower with larger value of P. Figure 4(b) shows the noise spectrum of inertial signal for different values of I, the value of P is fixed on −6 mHz/deg. It can be seen that the noise level is not variational with the value of I increases. It is also in agreement with the Fig. 3(e–h). The value of I cannot influence the sensitivity of closed-loop NMRG. Nevertheless, there are peaks for the noise spectrum in Fig. 4(b). The corresponding frequency of peaks increase with the value of I increases. It is also known from Fig. 3(e–h) that system will oscillate before it response to next resonant state. The bigger the value of I is, the stronger the system oscillates. Thus, if the frequency of noise is same with the oscillating frequency of step response for special P, I parameters, there will appear a peak on the noise spectrum.

**Figure 5:**
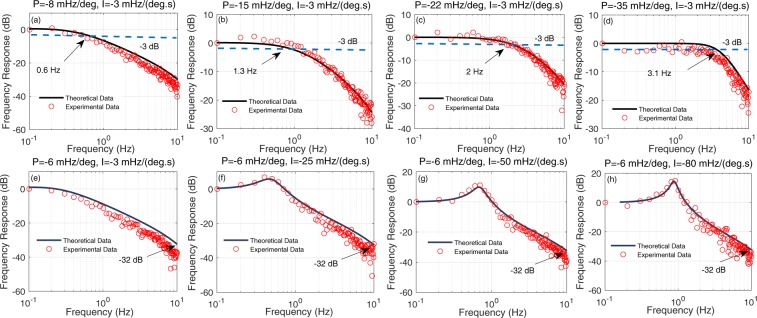
The frequency response of closed-loop NMRG system for various values of P, I. For (**a**–**d**), the value of I is fixed on −3 mHz/(deg.s). For (**e**–**h**), the value of P is fixed on −6 mHz/deg. The theoretical data are depicted by black lines, calculated through the close-loop transfer function $H(s)$. The red circles represent the experimental data.

Figure 5(a–d) show the frequency response of closed-loop NMRG for different values of P, with I fixed on −3 mHz/(deg.s). We obtain the frequency response through sweeping the frequency of a 3 nT oscillating magnetic field along *z* direction and measuring the corresponding amplitude of output signal of closed-loop NMRG experimentally, namely the amplitude of inertial signal. The red open circles represent the experimental data. The theoretical curves of frequency response are calculated via the transfer function *H*(*s*), depicted by the black solid line. The 3 dB bandwidth of different conditions have been illustrated. The measured bandwidths vary from 0.6 Hz to 3.1 Hz when the value of P increases from −8 mHz/deg to −35 mHz/deg. This is also corresponding to the quantative conclusion obtained in Fig. 3. The bandwidth of closed-loop NMRG depends on the value of P.

Figure 5(e–h) show the frequency response of closed-loop NMRG for different values of I, with P fixed on −6 mHz/deg. The value of I is −3 mHz/(deg.s), −25 mHz/(deg.s), −50 mHz/(deg.s), −80 mHz/(deg.s) for Fig. 5(e–h) respectively. It can be seen that the amplitude of output signal all decrease to −32 dB when the frequency of oscillating magnetic field reach to 10 Hz whatever the value of I is. Thus, we conclude that the bandwidth of closed-loop NMRG is not influenced by the value of I. Due to the oscillation of system for large value of I, there also have a peak on the curve of frequency response. When the frequency of 3 nT oscillating magnetic field along *z* axis is resonant with the oscillation frequency of step response with same values of P, I, there will appear a peak on the curve of frequency response. The amplitude and corresponding frequency of the peak increase with the value of I increases for the same value of P.

## Conclusion

In conclusion, we have demonstrated a closed-loop NMRG system experimentally and also constructed a theoretical model for the closed-loop NMRG system. We describe the dynamics of nuclear spins near the resonant point (i.e. working point) through Bloch equation. Then we solve the differential equation via Laplace transform and obtain the transfer function of open-loop NMRG system. Based on the open-loop NMRG system and automatic control theory, we obtained the transfer function of closed-loop system. The theoretical curves are obtained through the transfer function and MATLAB code. In order to study the influence of closed-loop control parameters for the performance of NMRG, we observed the step response, frequency response, noise spectrum experimentally for different closed-loop control parameters, which match very well with the theoretical results. We find that the value of closed-loop parameter P determine the bandwidth of NMRG and I has a optimal value when the value of P is fixed. The bandwidth and sensitivity of NMRG cannot be improved at the same time. The set of closed-loop control parameters depend on the concrete applications of NMRG.
